# Influence of Cultivar and Turbidity on Physicochemical Properties, Functional Characteristics and Volatile Flavor Substances of Pomelo Juices

**DOI:** 10.3390/foods12051028

**Published:** 2023-02-28

**Authors:** Jiajia Chen, Wenshan Luo, Lina Cheng, Jijun Wu, Yuanshan Yu, Lu Li, Yujuan Xu

**Affiliations:** 1 Sericultural & Argi-Food Research Institute, Guangdong Academy of Agricultural Sciences/Key Laboratory of Functional Foods, Ministry of Agriculture and Rural Affairs/Guangdong Key Laboratory of Agricultural Products Processing, No. 133 Yiheng Street, Dongguanzhuang Road, Tianhe District, Guangzhou 510610, China; 2 College of Food Science and Technology, Guangdong Ocean University, Zhanjiang 524088, China; 3 Guangdong Laboratory for Lingnan Modern Agriculture and China, Guangzhou 510642, China

**Keywords:** pomelo cultivars, pomelo juices, physicochemical characteristics, functional properties, volatile substances

## Abstract

In this study, the influences of pomelo cultivars on physicochemical properties, functional characteristics, and volatile compounds of juices were investigated. Among these six varieties, the highest juice yield (73.22%) was obtained in grapefruit. Sucrose and citric acid were the main sugar component and organic acid of pomelo juices, respectively. The results showed that the *cv.* Pingshanyu pomelo juice and grapefruit juice had the highest sucrose (87.14 g L^−1^, 97.69 g L^−1^) and citric acid content (14.49 g L^−1^, 13.7 g L^−1^), respectively. Moreover, the naringenin was the main flavonoid of pomelo juice. Additionally, the total phenolics, total flavonoids, and ascorbic acid concentrations of grapefruit and *cv.* Wendanyu pomelo juice were higher than those of other varieties of pomelo juices. Furthermore, 79 volatile substances were identified from the juices of six pomelo cultivars. Hydrocarbons were the predominant volatile substances, and the limonene was the characteristic hydrocarbon substance of pomelo juice. In addition, the pulp content of pomelo juice also presented great effects on its quality and volatile compounds composition. Compared to low pulp juice, the corresponding high pulp juice had higher sucrose, pH, total soluble solid, acetic acid, viscosity, bioactive substances and volatile substances. The effects of cultivars and variation in turbidity on juice are highlighted. It is useful for pomelo breeders, packers and processors to understand the quality of the pomelo they are working with. This work could provide valuable information on selecting suitable pomelo cultivars for juice processing.

## 1. Introduction

Fruits contain various bioactive compounds, such as flavonoids, phenolic acids, carotenoids, vitamins and phytoestrogens, which endow fruits with strong antioxidant activity, reduce symptoms of metabolic syndrome and improve heart disease [1]. Fruits are commonly eaten fresh, while many fruits needed to be processed to extend their shelf life. At present, the main types of processed fruit products are wines, vinegars, dried fruits, canned fruits, jams and juices [2]. Juice is an important element of a healthy diet, supplying essential nutrients [3]. The common types of juices on the market mainly include concentrated fruit juice, water extract fruit juice, dehydrated fruit juice, fermented fruit juices, powdered fruit juice, and not from concentrated (NFC) juice [4]. NFC juices are products that are obtained by pressing fruit, separating the pulp and debris to the required level, and then sterilizing and packaging it into containers for consumer use [5]. Among these juices, NFC juice is the most popular among consumers due to its natural fresh appearance, texture, lack of food additives and flavor [6]. However, the main sources of NFC juices were only apples, grapes and oranges [5], which caused difficulties in meeting the increasing demands of consumers. Therefore, NFC juices for other fruits need to be developed.

Pomelos, also known as pummelo (*Citrus grandis* (L.) Osbeck), are one of the three original species of citrus in the *Aurantioideae* subfamily under the *Rutaceae* family. It originated in Southeast Asia and has been cultivated in China for over 3000 years [7]. Pomelos are rich in bioactive substances, with antioxidant capacities, inflammatory, anticancer and antibacterial properties and preventative abilities regarding cardiovascular disease and diabetes [8]. It had been reported that native pomelo cultivars include *cv.* Deep Red, *cv.* Shatianyu, *cv.* Pingshanyu, *cv.* Yuhuanyu, *cv.* Guanxiyu, *cv.* Pingshanyu, *cv.* Cuixiangtianyu, *cv.* Changshanyu, etc. [9]. According to previous research, the cultivars of fruits have a great influence on their physicochemical properties [10]; Liu [11] found that the aroma-active compounds of fresh watermelon juice in five cultivars were significantly different. However, there are few studies about the effect of pomelo’s cultivars on its physicochemical characteristics [12].

Pomelo fruits are often consumed fresh or processed into juice, candy, jam, wine, and essential oils [13]. As an important value-added product, fruit juice is valued by consumers around the world. Among these fruit juices, grapefruit juice is one of the most common processed products of pomelo fruit, and is preferred by consumers because of its rich nutrients and unique taste [14], while other cultivars of pomelo juice are not commonly available on the market. It is well known that the active ingredients [10] and volatile compounds [15] are significantly varied with different fruit cultivars. Teleszko et al. [16] also found that a significant difference in phenolic content was observed among seven different cultivars of strawberry juices. However, most studies of pomelo juice mainly focus on extending the storage life of pomelo juice by using sterilization technology [17] and debittering pomelo juice to increase its acceptability [18]. The effect of the pomelo cultivar on the quality of pomelo juice has been less reported [19]. In addition, the pulp content of juice also had a great influence on its quality [20]. Harzallah et al. [21] suggest that the fig pulp content affects the antioxidant capacities of fig juices. Therefore, the aim of study was to evaluate the differences in physicochemical properties, functional characteristics, and volatile compounds in low pulp and high pulp pomelo juices of six cultivars.

## 2. Materials and Methods

### 2.1. Plant Material

Six cultivars of matured pomelo fruits, grapefruit (Taiwan (121°59′ N, 25°05′ E), China), *cv.* Guanxiyu (Zhangzhou City (117°64′ N, 24°52′ E), Fujian Province, China), *cv.* Wendanyu (Xiangyou County (118°67′ N, 25°37′ E), Fujian Province, China), *cv.* Liangpingyu (Liangping county (117°63′ N, 24°52′ E), Chongqing City, China), *cv.* Pingshanyu (Zhangzhou City (117°64′ N, 24°52′ E), Fujian Province, China) and *cv.* Shatianyu (Rong Country (110°57′ N, 22°89′ E), Guangxi Province, China) were collected from their main production areas in China. Pomelo plants used in this study were ripe, healthy, diseases free and grown in open fields. Each cultivar of pomelo was purchased in 10 kg amounts and stored at 4 °C.

### 2.2. Chemicals and Water Purification

Chromatography methanol and acetonitrile were purchased from Merck (Darmstadt, Germany). Other reagents (analytically pure) were purchased from Tianjin Damao Chemical Reagent Factory (Tianjin, China). Water was purified using a Milli-Q system (Bedford, MA, USA).

### 2.3. Preparation of the Pomelo Juices

**Figure 1:**
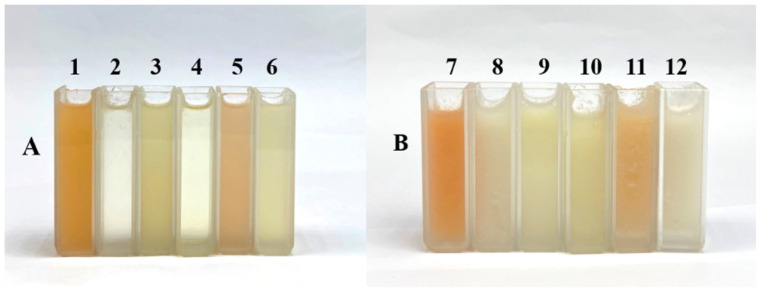
Images of LP (**A**) and HP (**B**) juices of different pomelo cultivars. Juice types: 1 and 7, grapefruit juice; 2 and 8, Guanxiyu pomelo juice; 3 and 9, Wendanyu pomelo juice; 4 and 10, Liangpinyu pomelo juice; 5 and 11, Pingshanyu pomelo juice; 6 and 12, Shatianyu pomelo juice.

The ripe pomelo fruits were manually peeled; only the pulp was used as the experimental material. The well-mixed pulps were randomly divided into two equal groups for the juicing treatments: one group was squeezed using a kitchen juicer (HR1889; Philips, China) through a filter (low pulp, LP), and the other group used a high speed blender (PBJ-S02E; Bestday, China) without a filter (high pulp, HP). The images of LP and HP pomelo juices were presented in Figure 1. Each cultivar of juices produced at least 3 bottles and was stored at −20 °C to reduce microbial contamination for further analysis.

### 2.4. Determination of Total Soluble Solid, pH and Juice Yield

The total soluble solid (TSS) of pomelo juices was determined by a digital refractometer (RP-101, Atago, Tokyo, Japan) at 20 °C and expressed as ‘Brix’. The pH values of pomelo juice samples were measured at room temperature using a pH meter (PB-10, Sartorius, Germany). Additionally, the pH meter was calibrated before measurement.

The pomelo pulp (M_2_) was squeezed using a juicer (HR1889; Philips, Shanghai, China). After centrifugation (high speed centrifuge, D3024R, scilogex, America) at 6000 rpm for 10 min, the supernatant was immediately collected and weighed (M_1_). The juice yield was calculated by the following formula:

$$\mathrm{juice}\;\mathrm{yield}\left(\%\right)=M_{1}/M_{2}\times 100$$

where M_1_ and M_2_ were the weights of the juice and pulp, respectively.

### 2.5. Color Analysis

Color analysis was performed in reflection mode using a colorimeter (Hunter Lab Co., Reston, VA, USA) at 25 °C. The colorimeter was calibrated with the white standard disk. The *L*, *a*, and *b* expressed brightness value, green to red values, and blue to yellow values, respectively. Three measurements were measured and the results were averaged.

### 2.6. Determination of Total Flavonoids

Total flavonoid content was assayed according to the modified method of Han et al. [22]. The rutin was used as the standard substance for the quantification of total flavonoids content (concentration range of 0–0.9 mg mL^−1^). One mL juice sample was mixed with 0.5 mL methanol hydrochloride (MeOH : HCl : H_2_O = 80:1:19). Then, 0.3 mL 5% sodium nitrite solution was added to 1 mL mixture and reacted for 6 min. The reaction product was mixed with 0.5 mL 10% aluminum nitrate solution, and reacted for 6 min. Four mL 1 mol L^−1^ sodium hydroxide solution was added into the mixture and it was left for 15 min. After centrifugation at 3000 rpm for 10 min, the absorbance of the supernatant was measured by UV-visible spectrophotometer (UV1800; Shimadzu Co., Ltd., Tokyo, Japan) at 505 nm. The total flavonoids content of the sample was expressed in mg rutin equivalent (RE) per liter of sample.

### 2.7. Determination of Total Phenolics

The total phenolic content was determined using the method described by Ghafoor et al. [23], with a minor modification. The juice samples were mixed with methanolic hydrochloride (1:12.5) and two mL Folin–Ciocalteu reagent and 2 mL 10% sodium carbonate were added into 1 mL mixture, respectively. Then, the reaction was protected from light for 1 h. Absorbance was measured at 760 nm. The total phenolics content was presented as mg of gallic acid (0–0.06 mg mL^−1^) equivalents (GAE) per liter of sample. The gallic acid standards curve was y=29.629x−0.0031 (R^2^ = 0.9991).

### 2.8. HPLC Analysis of Sugar Component, Organic Acid and Flavonoid Composition

**Figure 2:**
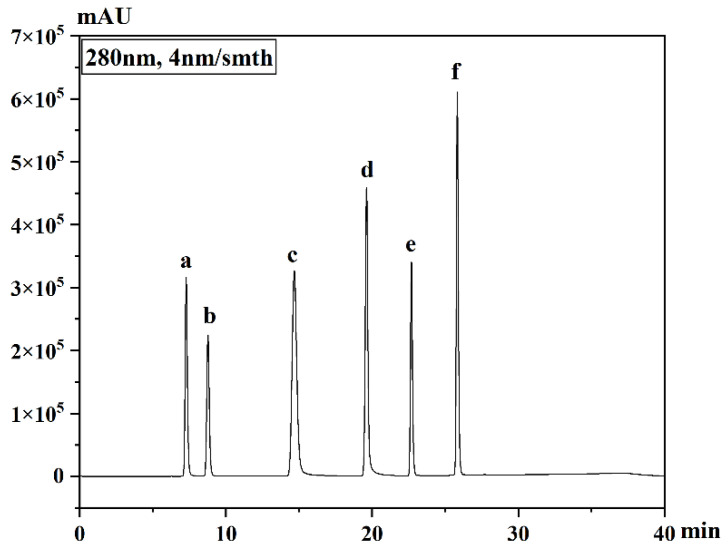
The flavonoid separation chromatogram of standards. a, Narirutin; b, Naringin; c, Hydroxynaringenin; d, Naringenin, e, Sinensetin; f, Hesperidin.

HPLC (LC-20at; Shimadzu Co., Ltd., Tokyo, Japan) was used to analyze sugar components (fructose, glucose and sucrose) [24], organic acid [25] and flavonoid compound [26]. The sugar component involved acetonitrile: water (70:30, *v/v*) as the mobile phase at a flow rate of 1.0 mL min^−1^ with a Shodex packed column (AsahipakNH2P-504E, 4.6 mm × 250 mm) and quantified by ELSD detector. The organic acids were separated by WondaSil packed column (C18 primec N5020-39103, 4.6 mm × 250 mm) under the condition of 30 °C and 0.1 mol L^−1^ (NH_4_)_2_HPO_4_. The detection wavelength of organic acid was 245 nm. The Agilent packed column (ZORBAXE eclipse Plus C18, 4.6 mm × 250 mm) was used to separate flavonoid compounds using the gradient elution methanol (0–10 min, methanol: water = 40:60; 10–20 min, methanol: water = 60:40; 20–30 min, methanol: water = 80:20; 30–40 min, methanol = 100) and quantified by photodiode array detector at the wavelength of 280 nm. The injection volume was 10 μL. The flavonoid separation chromatogram of standards is presented in Figure 2. All the flavonoid compounds were identified according to the retention time, and the concentration was calculated based on the external standard curve of the standard.

### 2.9. Determination of Viscosity

The determination of the viscosity of the sample was carried out using a rheometer (AR1500EX, TA, New Castle, DE, USA) according to a previous study [27]. A cone/plate sensor system (CP25-2) with a gap width of 6 cm was used to measure the viscosity of the sample. The temperature in the gap of the rheometer was 25 ± 0.1 °C. Flow curves and apparent viscosities were recorded using shear rates γ in the range of 0–100 s^−1^. The sample remained stationary in the sensor unit for 3 min.

### 2.10. Analysis of Volatile Compounds

The volatile compounds were extracted and measured using the headspace-SPME GC-MS method as described by Goh [28] with some modifications. Adding 5 mL juice and 1.8 g NaCl in the screw injection bottle promoted the volatilization of aroma components. The bottle was sealed with polytetrafluoroethylene gasket and balanced on the magnetic agitator at 40 °C for the 15 min, then absorbed by a SPME fiber (Supelco, Bellefonte, PA, USA) coated with 50/30 μm Divinylbenzene/Carboxen/Polydimethylsiloxane (DVB/CAR/PDMS) at 40 °C for 40 min. The chromatographic column was elastic quartz capillary column HP-5 (5% diphenyl-95% dimethlpolysiloxane, 30 m × 0.25 mm × 0.25 µm). The helium gas flow rate and inlet temperature were 1.2 mL/min and 250 °C, respectively. The temperature program was as follows: 40 °C for 3 min, heating to 160 °C by 3 °C/min and holding at 160 °C for 2 min, then heating from 160 °C to 220 °C at 8 °C/min, and holding at 220 °C for 3 min. The chromatograms and mass spectra were evaluated with the GC–MS Postrun Analysis software (GC-MS-QP2010, SHIMADZU, Tokyo, Japan). The compounds were tentatively identified by comparing their mass spectra with those in the data system library (NIST14). Quantitative analysis was carried out using the internal standard method. The internal standard is cyclohexanone (10 μL, 100 mg L^−1^). The concentrations of volatile components were expressed as mg mL^−1^. Three replicates were used in each sample.

### 2.11. Statistical Analysis

The plant material contained 3 biological replicates, where each replicate had 9 harvested fruits. All results were expressed as average value ± standard deviation (SD) of three replicates. The data were analyzed by one-way ANOVA of SPSS 26.0 software (Chicago, IL, USA). Significant differences among the samples were calculated using one-way ANOVA followed by Duncan’s multiple-range test at the 5% level (*p* < 0.05).

## 3. Results and Discussion

### 3.1. Effect of Pomelo Cultivars on Juice Yield, Total Soluble Solid, pH of Juices

In the food industry, fruits with a high juice yield could reduce the cost of producing NFC juices to a certain extent [5]. Hence, the impact of pomelo cultivars on juice yield was studied and the results were presented in Table 1. The juice yields of six pomelo cultivars were significantly different. Among these six pomelo cultivars, grapefruit had the highest juice yield (73.22%), which was 28.04%, 11.73%, 22.18%, 26.36% and 38.06% higher than that of *cv*. Guanxiyu, *cv*. Wendanyu, *cv*. Liangpinyu, *cv*. Pingshanyu and *cv*. Shatianyu, respectively. The lowest juice yield was from *cv*. Wendanyu. There was little difference in the juice yield between *cv.* Guanxiyu and *cv.* Pingshanyu. Ivanova et al. [15] reported a juice yield of 70.12% for grapefruit, which was similar to the result of this study. In addition, the juice yield of *cv.* Wendanyu was lower than the conclusion reached by Di Matteo et al. [29] in their analysis of lemon juice. The results showed that pomelo cultivars significantly affect the juice yield.

Sugar components (fructose, glucose, sucrose) played a major role in the taste of juice [30]. As shown in Table 2, sucrose was the major sugar in pomelo juices, followed by glucose, which was consistent with the report of orange juices [31]. Grapefruit juice obtained the highest fructose and glucose contents, respectively, while the lowest contents of fructose and glucose content were found in *cv*. Shatianyu juice. Moreover, the fructose content of all pomelo juices was much lower than Marsh grapefruit juice reported by Zheng et al. [32]. The sucrose concentrations of most HP pomelo juices were higher than those of the corresponding LP juices, while the opposite phenomenon was found in glucose and fructose. This phenomenon could be attributed to the fact that the pomelo pulps were obtained under high-speed extrusion, which ruptured the cell walls and released more sucrose from the pectin [33]. These results suggested that the varietal differences led to differences in the sugar component concentrations of pomelo juices.

It had been reported that the TSS content in juices in general did depend on the cultivars [34]. The TSS content for these juices ranged from 10.55 to 13.30 ‘Brix’, which was much higher than for the white pomelo juices reported by Cheong et al. [19]. Moreover, in addition to HP Liangpinyu juice (HPLU), the TSS contents of most HP juices were always higher than those of LP pomelo juices. The taste of the juices was great affected by pH value, and a significant difference was found in the pH value among different pomelo juices. The pH value of the LP juice of the same pomelo cultivar was lower than that of HP ones. Grapefruit juice had the lowest pH value (3.24), while the pH of HP Liangpinyu juices (HPLU) and HP Shatianyu juices (HPSU) were the highest (4.75). These results indicated that the TSS content, pH value, glucose and sucrose concentration of the HP pomelo juice of most pomelo cultivars were higher than those of LP pomelo juices. Additionally, there was wide diversity between cultivars.

### 3.2. Effect of Pomelo Cultivars on Organic Acids Composition of Pomelo Juices

Organic acids were an important class of compounds in fruit juices. A previous study found that malic acid, citric acid, and oxalic acids are the main organic acids of citrus fruits [35]. As shown in Table 3, oxalic acid, tartaric acid, acetic acid, malic acid, citric acid and succinic acid were identified in pomelo juices. Malic acid, ascorbic acid, acetic acid, and citric acid were presented in all pomelo juices. Among these four organic acids, citric acid was the main organic acid of pomelo juices, accounting for 59.59–87.03% of the total organic acid; the same result was also found in orange juice [36]. The highest citric acid content was observed in HP grapefruit juice (HPGT, 14.49 g L^−1^). The organic acid concentration of LP juice of most pomelo cultivars was higher than those of HP juices. A similar result was also found in winter melon juice that the LP juice (2.5 g kg^−1^) exhibited higher malic acid contents compared to HP juice (1.4 g kg^−1^) [37]. In general, there was a positive correlation between pH and organic acid. Most LP pomelo juices exhibited lower total organic acid content in comparison with the corresponding HP pomelo juices, which might be the reason that the pH of the LP pomelo juices was lower than that of HP pomelo juices.

### 3.3. Effect of Pomelo Cultivars on Color and Viscosity of Juices

The color parameters (*L*, *a*, *b*) of different pomelo juices were also detected. The color of the juice was decisive in juice acceptance by the consumers as it reflected the quality, safety, and nutritional values [36]. The results are presented in Table 4. The colors of the pomelo juices from different cultivars were markedly different. In general, the juices had a high brightness when the *L* value above 30 [1]. The *L* values of all pomelo juices were higher than 30, and the *L* values of most HP pomelo juices were higher than those of corresponding LP pomelo juices, revealing that all pomelo juices had a strong brightness and most HP pomelo juices showed a higher brightness. Furthermore, the juice color was affected by the fruit characteristics, such as cultivar, ripening, pulps and growth conditions [38]. The *a* and *b* values of grapefruit juices, as well as *cv.* Pingshanyu juices, had positive value, indicating that the color of them were strong reddish yellow, while the *a* and *b* values of *cv*. Guanxiyu juices showed negative values, which revealed that the yellow-green color was found in *cv.* Guanxiyu juices. Hence, it could be concluded that the difference in the b values of the guava juices were mainly dependent on the cultivar. The results were consistent with the report about sugarcane juice [39].

**Figure 3:**
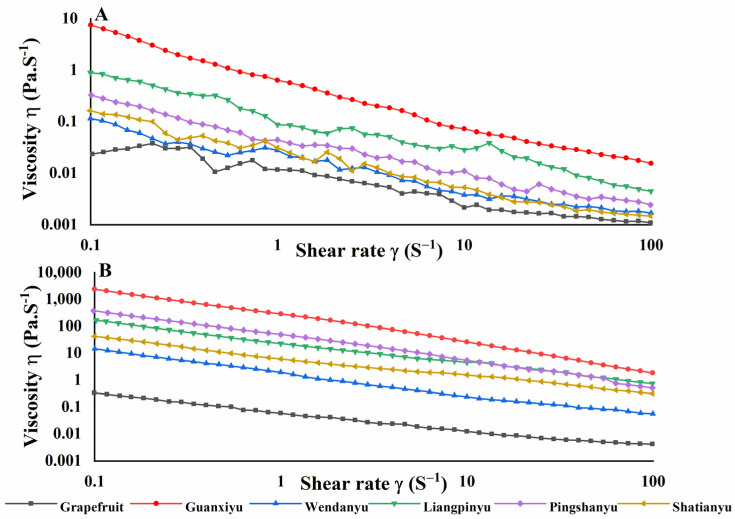
Effects of cultivars on viscosity of pomelo juices. (**A**) LP juice; (**B**) HP juice.

The viscosity of the juices could reflect the stability of the juices [40]. The viscosity of pomelo juices of different cultivars is shown in Figure 3. As the shear rate increased the viscosity of pomelo juices gradually decreased, which was in line with a non-Newtonian fluid. The viscosity of the HP pomelo juices was higher by contrast with the LP pomelo juices, probably due to the lower pulp content in the juices [41]. The highest apparent viscosities of LP and HP pomelo juices were found in *cv.* Guanxiyu, while the grapefruit juices had the lowest. The level of TSS content markedly, non-linearly affected the apparent viscosity of non-Newtonian fluids [27], which could partly explain the differences in the viscosities of different cultivars of pomelo juices. The same results were also observed in apple juice [34].

### 3.4. Effect of Pomelo Cultivars on the Contents of Total Phenolics (TP), Total Flavonoids (TF) and Ascorbic Acid of Juice

**Figure 4:**
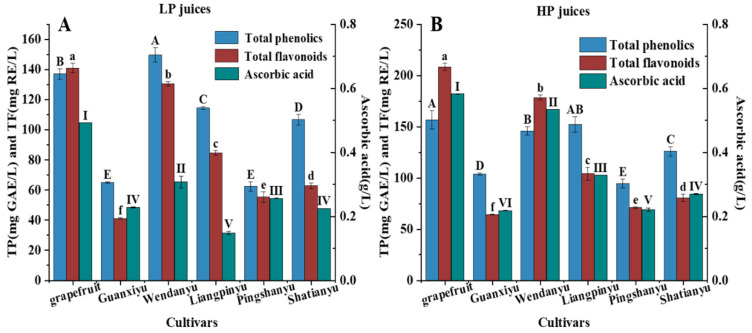
Effects of cultivars on TP, TF and ascorbic acid content of pomelo juices. (**A**) LP juice; (**B**) HP juice; data are expressed as means ± standard deviation (*n* = 3); different letters indicate significantly different means at *p* < 0.05.

Citrus fruits are rich in phenolics, flavonoids and ascorbic acid, which have the capacities of reducing the risk of inflammation, mutagenesis and carcinogenesis [42]. Many scholars have found that the TP, TF and ascorbic acid concentrations in citrus fruits are greatly impacted by their cultivar [43]. The concentrations of TP, TF and ascorbic acid in different cultivars’ pomelo juices are presented in Figure 4. In our study, the differences in ascorbic acid contents varied from 0.15 g L^−1^ (LP Liangpinyu juice) to 0.58 (HPGT) g L^−1^. Compared to LP pomelo juices, most HP pomelo juices had higher contents of TP, TF and ascorbic acid, which was consistent with the result in apple juice, where the TP concentration of HP juice was higher than that of LP juice [44]. Among these pomelo fruit juices, HPGT obtained the highest TP (156.75 mg GAE L^−1^), TF (208.37 mg RE L^−1^), and ascorbic acid (0.58 g L^−1^) contents, while the lowest concentrations of TP, TF and ascorbic acid were all found in the LP Guanxiyu juice (LPGU). The TP and TF content of the HPGT juice were 2.28- and 5.07-fold higher than those of LPGU juices, respectively. This finding is similar to the previous study on pineapple juice [45]. The cultivars’ diversity resulted in different levels of TP, TF and ascorbic acid in pomelo juices, and these three bioactive substances were generally higher in HP juices than LP juices.

### 3.5. Effect of Pomelo Cultivars on Flavonoid Composition of the Juice

Flavonoids compounds exist widely in citrus fruits, and they are the main reason for the bitter taste and bioactive properties of citrus fruit juices [46]. Therefore, the flavonoid composition of different cultivars’ pomelo juices was investigated, and the results were presented in Table 5. The narirutin, naringin, hydroxynaringenin, naringenin, sinensetin and hesperidin concentrations of pomelo juices were measured. Only the narirutin and naringenin were detected in all the pomelo juices, which were the main bitter substances of pomelo juices [47]. Moreover, these flavonoids contents of most HP juices were higher than those of LP juice, which might be due to these flavonoids mainly existing in the pulp. HPGT juice obtained the highest concentration of flavonoid composition, which was consistent with the result in Figure 4. The highest concentrations of narirutin and naringin were observed in HPLU and LP grapefruit juice (LPGT), respectively. Among these pomelo juices, the HPLU exhibited higher contents of narirutin and naringenin, indicating that the taste of HPLU was more bitter. Kim et al. also found that the HP citrus juice had higher contents of narirutin [48]. Meanwhile, sinensetin was found in grapefruit juices and *cv.* Wendanyu juices, while the hesperidin and hydroxynaringenin were only detected in *cv.* Liangpingyu juice and grapefruit juice, respectively. These results indicated that the contents and types of flavonoid compounds of pomelo juices were significant influenced by their cultivars. Therefore, *cv.* Guanxiyu juice was much better for consumption for people in comparison with other pomelo juices.

### 3.6. Effect of Pomelo Cultivars on Volatile Compounds of the Juice

**Figure 5:**
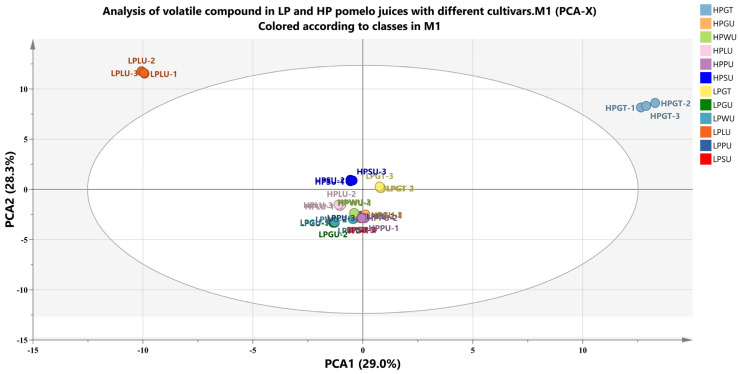
Principal component analysis (PCA) of volatile substances of different pomelo juices. LPGT, LP grapefruit juice; LPGU, LP Guanxiyu juice; LPWU, LP Wendanyu juice; LPLU, LP Liangpinyu juice; LPPU, LP Pingshanyu juice; LPSU, LP Shatianyu juice; HPGT, HP grapefruit juice; HPGU, HP Guanxiyu juice; HPWU, HP Wendanyu juice; HPLU, HP Liangpinyu juice; HPPU, HP Pingshanyu juice; HPSU, HP Shatianyu juices.

Aroma, composed of a large number of volatile compounds, is one of the sensory properties of fruit juice and has a great effect on the acceptance of fruit juice [49]. As shown in Figure 5, the correlation of volatile compounds of different pomelo juices was evaluated by principal component analysis (PCA). The first two principal components extracted accounted for 57.3% (PC1 29.0%, PC2 28.3%). The scatter points corresponding to the samples of the 12 groups showed clustering with each other within the groups, indicating that the repeatability within the groups was relatively good. The LPPU, LPSU, LPWU, HPGU, HPWU and HPPU were highly clustered, and were located in the negative quadrant of PCA1 and the positive quadrant of PCA2, while for other samples there was no aggregation between groups, revealing that there were differences in the flavor composition of pomelo juice of different cultivars.

**Figure 6:**
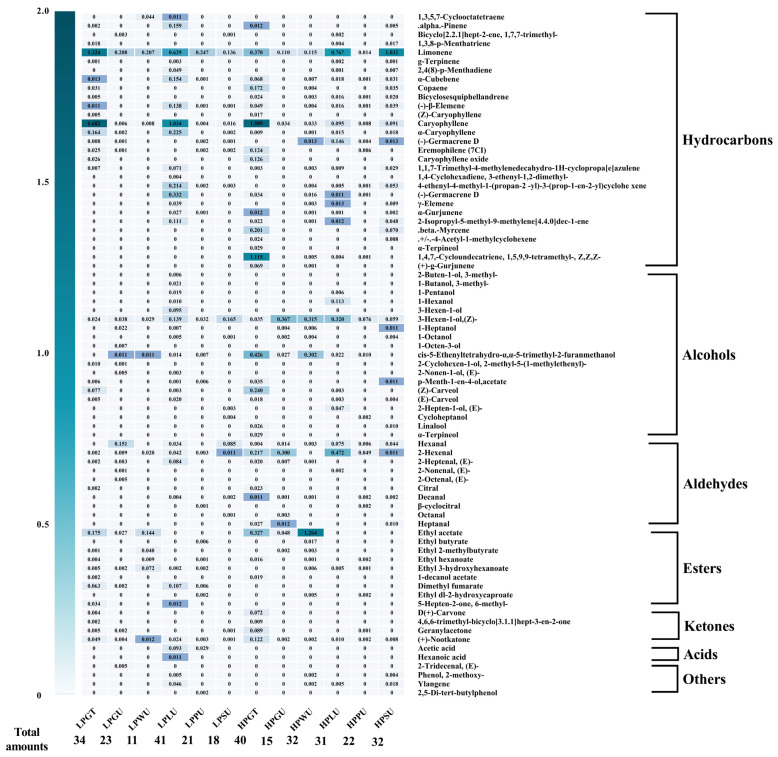
The relative content of volatile compounds of different pomelo juices. For the same substance, the whiter its color, the lower its content; the bluer its color, the higher its content. LPGT, LP grapefruit juice; LPGU, LP Guanxiyu juice; LPWU, LP Wendanyu juice; LPLU, LP Liangpinyu juice; LPPU, LP Pingshanyu juice; LPSU, LP Shatianyu juice; HPGT, HP grapefruit juice; HPGU, HP Guanxiyu juice; HPWU, HP Wendanyu juice; HPLU, HP Liangpinyu juice; HPPU, HP Pingshanyu juice; HPSU, HP Shatianyu juices.

In order to further study the volatile compounds composition of different pomelo juices, the relative contents of volatile compounds in different pomelo juices were analyzed (Figure 6). A total of 79 volatile compounds were detected, including thirty hydrocarbons, twenty alcohols, ten aldehydes, eight esters, five ketones, two acids and four others. The most types of volatile substances were detected in LPLU (41), followed by HPGT (40). Hydrocarbons accounted for the highest proportion in volatile compounds of pomelo juices. Among these detected hydrocarbons, limonene and caryophyllene were determined in all pomelo juices. Limonene was an important citrus aroma compound and one of the main components of the juice, which was consistent with previous reports in the studies [50]. LPGT exhibited the highest relative content of limonene (1.257 mg mL^−1^); almost half of the LPGT volatiles were contributed by limonene, followed by HPSU (0.979 mg mL^−1^). In addition, the caryophyllene relative content of LPGT was the highest among these pomelo juices (1.682 mg mL^−1^), which was mainly derived from citrus peel and had a cultivar of important pharmacological activities [51]. With respect to alcohols, the relative content of alcohols in HP juice was higher than those in LP juice. 3-hexen-1-ol, (Z)- was identified in all pomelo juices, which was the dominant alcohol of pomelo juice and had a strong aroma of grass. The highest relative concentration of 3-hexen-1-ol, (Z)- was found in HPGU (0.367 mg mL^−1^), whereas α-terpincol was only detected in HPGT juice. 2-buton-1-ol, 3-methyl-, 1-butanol, 3-methyl- and 3-hexen-1-ol were only found in LPLU. Regarding aldehydes, more categories of aldehydes were measured in HP juices than in LP juices and the HP juices also had a higher relative level of aldehyde. 2-hexenal was the major aldehyde of pomelo juice, which was measured in all samples. 2-octenal, (E)- was only detected in LPGU. However, none of the esters were measured in any of the pomelo juices. According to previous research, ethyl acetate was the main ester in juice and it had a fruity flavor and gave the juice its pleasant aroma [52]. The ethyl acetate was only detected in grapefruit, *cv*. Guanxiyu and *cv*. Wendanyu pomelo juices, and the highest content of ethyl acetate was found in HPWU (1.264 mg mL^−1^). For other volatile substances, the pomelo-flavored (+)-nootkatone was detected in all samples and HPGT had the highest content of (+)-nootkatone. The above results suggested that the differences in volatile substances in different cultivars of pomelo juice were mainly focused on their relative contents and the amounts, and the HP juice was mostly superior to the LP juice in terms of amounts and relative content of volatile substances. HPGT and LPLU have a stronger aroma and flavor profile.

## 4. Conclusions

The aim of this study was to evaluate the effects of pomelo cultivar and turbidity on physicochemical properties, functional characteristics, and volatile compounds of juices. The findings of this research showed that most of the HP juices had higher sucrose content, pH, total soluble solid, acetic acid concentration, viscosity, bioactive substances contents and volatile substances amounts compared to corresponding LP pomelo juices. Sucrose is the dominant sugar in pomelo juices, occupying 56.20–93.12% of the total sugars. HPGT had the highest levels of TP (156.75 mg RE L^−1^), TF (208.37 mg GAE L^−1^), ascorbic acid (0.58 g L^−1^), citric acid (13.7 g L^−1^) and naringin (1.11 g L^−1^). In addition, the pomelo cultivars and turbidity had a great impact on the types and concentrations of volatile substances in juice. The present contribution revealed that a total of 79 volatile substances were identified from different pomelo juices. Hydrocarbons were the main volatile compounds, in particular limonene and caryophyllene, which were the main contributors to the aroma. LPLU showed higher volatile substance types (41) in comparison with other pomelo juices. In combination, it could be concluded that the *cv.* Liangpinyu and grapefruit cultivars were suitable for processing into juice. This study provided a basis for the processing of different types of pomelo fruit juices and valuable information for pomelo quality breeding and consumer guidelines.

## Figures and Tables

**Table 1:** Effect of cultivars on the yield of pomelo juice.

Cultivars	Grapefruit	Guanxiyu	Wendanyu	Liangpinyu	Pingshanyu	Shatianyu
Juice yield (%)	73.22 ± 2.01 ^a^	52.69 ± 3.09 ^c^	64.63 ± 1.32 ^b^	56.98 ± 3.66 ^c^	53.92 ± 3.51 ^c^	45.35 ± 3.11 ^d^

Data are expressed as means ± standard deviation (*n* = 3); different letters indicate significantly different means at *p* < 0.05.

**Table 2:** Effects of cultivars on sugar component, pH, and total soluble solids of pomelo juices.

Types	Cultivars	Sugar Component (g L^−1^)			pH	Total Soluble Solids (‘Brix’)
		Fructose y = 791724x – 56967 (R² = 0.998)	Glucose y = 520381x – 123387 (R² = 0.991)	Sucrose y = 573278x − 77121 (R² = 0.995)		
LP juices	Grapefruit	14.02 ± 0.41 ^a^	27.15 ± 0.55 ^b^	40.49 ± 0.98 ^j^	3.24 ± 0.00 ^j^	11.4 ± 0.00 ^h^
	Guanxiyu	7.74 ± 0.26 ^c^	17.64 ± 0.15 ^c^	81.01 ± 1.74 ^d^	3.39 ± 0.01 ^h^	12.26 ± 0.04 ^f^
	Wendanyu	6.77 ± 0.28 ^d^	17.11 ± 0.69 ^c^	57.76 ± 0.73 ^h^	3.58 ± 0.01 ^f^	11.75 ± 0.00 ^j^
	Liangpinyu	3.15 ± 0.20 ^f^	9.91 ± 0.48 ^f^	62.28 ± 0.80 ^g^	4.74 ± 0.011 ^b^	12.49 ± 0.00 ^l^
	Pingshanyu	5.35 ± 0.026 ^e^	14.33 ± 0.34 ^e^	87.14 ± 0.39 ^b^	3.75 ± 0.01 ^e^	12.49 ± 0.03 ^e^
	Shatianyu	1.47 ± 0.12 ^g^	5.22 ± 0.045 ^g^	83.12 ± 1.73 ^c^	4.74 ± 0.011 ^b^	11.86 ± 0.00 ^g^
HP juices	Grapefruit	12.88 ± 0.81 ^b^	28.57 ± 1.39 ^a^	53.18 ± 0.15 ^i^	3.31 ± 0.00 ^i^	11.7 ± 0.00 ^i^
	Guanxiyu	7.19 ± 0.21 ^d^	17.45 ± 0.42 ^c^	84.14 ± 0.84 ^c^	3.52 ± 0.01 ^g^	13.18 ± 0.021 ^b^
	Wendanyu	5.66 ± 0.081 ^e^	15.48 ± 0.31 ^d^	76.87 ± 0.31 ^e^	3.85 ± 0.01 ^d^	12.68 ± 0.00 ^c^
	Liangpinyu	2.93 ± 0.10 ^f^	9.12 ± 0.76 ^f^	69.95 ± 0.86 ^f^	4.75 ± 0.01 ^a^	10.55 ± 0.00 ^k^
	Pingshanyu	5.22 ± 0.27 ^e^	13.56 ± 1.02 ^e^	97.69 ± 1.06 ^a^	3.96 ± 0.01 ^c^	13.30 ± 0.01 ^a^
	Shatianyu	1.53 ± 0.18 ^g^	4.59 ± 0.078 ^g^	82.92 ± 0.94 ^c^	4.75 ± 0.01 ^a^	12.59 ± 0.00 ^d^

Data are expressed as means ± standard deviation (*n* = 3); different letters indicate significantly different means at *p* < 0.05.

**Table 3:** Effects of cultivars on organic acids of pomelo juices.

Types	Cultivars	Oxalic Acid (g L^−1^) y = 10499663.84x + 88080.34247 (R^2^ = 0.999)	Tartaric Acid (g L^−1^) y = 1068533.055x − 32284.32329 (R^2^ = 0.999)	Malic Acid (g L^−1^) y = 454821x + 1516.4 (R^2^ = 0.999)	Acetic Acid (g L^−1^) y = 376758x + 5878.5 (R^2^ = 0.999)	Citric Acid (g L^−1^) y = 619376x + 11163 (R^2^ = 0.999)	Succinic Acid (g L^−1^) y = 353408x − 26483 (R^2^ = 0.999)
LP juices	Grapefruit	-	0.85 ± 0.55 ^a^	0.57 ± 0.01 ^g^	0.66 ± 0.03 ^g^	14.49 ± 0.01 ^a^	-
	Guanxiyu	0.16 ± 0.04 ^e^	0.45 ± 0.15 ^e^	0.69 ± 0.05 ^f^	0.77 ± 0.04 ^efg^	10.96 ± 1.59 ^b^	-
	Wendanyu	-	0.48 ± 0.69 ^e^	0.98 ± 0.05 ^cd^	1.47 ± 0.06 ^ab^	7.71 ± 0.13 ^c^	0.71 ± 0.08 ^b^
	Liangpinyu	-	0.28 ± 0.48 ^f^	0.81 ± 0.041 ^e^	1.51 ± 0.01 ^ab^	5.57 ± 0.41 ^d^	0.64 ± 0.09 ^b^
	Pingshanyu	0.22 ± 0.22 ^c^	-	1.11 ± 0.06 ^a^	0.91 ± 0.39 ^efg^	7.85 ± 0.72 ^c^	-
	Shatianyu	0.21 ± 0.00 ^d^	-	0.91 ± 0.03 ^d^	1.30 ± 0.27 ^bcd^	5.78 ± 0.39 ^d^	0.94 ± 0.02 ^a^
HP juices	Grapefruit	-	0.73 ± 0.029 ^b^	0.45 ± 0.03 ^h^	0.69 ± 0.18 ^fg^	13.7 ± 2.15 ^a^	-
	Guanxiyu	0.21 ± 0.01 ^d^	0.56 ± 0.00 ^d^	0.71 ± 0.01 ^f^	1.09 ± 0.13 ^cde^	10.32 ± 1.36 ^b^	-
	Wendanyu	-	0.61 ± 0.01 ^c^	1.08 ± 0.03 ^ab^	1.39 ± 0.1 ^bc^	6.94 ± 0.03 ^cd^	0.70 ± 0.02 ^b^
	Liangpinyu	-	0.47 ± 0.04 ^e^	0.53 ± 0.11 ^g^	1.78 ± 0.23 ^a^	5.61 ± 0.1 ^d^	0.44 ± 0.02 ^c^
	Pingshanyu	0.29 ± 0.01 ^a^	-	1.02 ± 0.01 ^bc^	1.01 ± 0.16 ^def^	6.78 ± 0.41 ^cd^	-
	Shatianyu	0.27 ± 0.01 ^b^	-	0.71 ± 0.01 ^f^	1.61 ± 0.06 ^ab^	6.31 ± 0.04 ^cd^	0.65 ± 0.06 ^b^

Data are expressed as means ± standard deviation (*n* = 3); different letters indicate significantly different means at *p* < 0.05.

**Table 4:** Effects of cultivars on color indexes of pomelo juices.

Types	Cultivars	*L*	*a*	*b*
LP juices	Grapefruit	33.73 ± 0.05 ^h^	1.64 ± 0.02 ^b^	3.43 ± 0.02 ^a^
	Guanxiyu	33.24 ± 0.05 ^i^	−0.42 ± 0.02 ^e^	−0.82 ± 0.02 ^f^
	Wendanyu	37.10 ± 0.02 ^f^	−1.55 ± 0.02 ^j^	0.42 ± 0.02 ^e^
	Liangpinyu	37.92 ± 0.02 ^e^	−2.13 ± 0.02 ^k^	1.68 ± 0.03 ^b^
	Pingshanyu	31.86 ± 0.02 ^k^	1.52 ± 0.03 ^c^	0.98 ± 0.04 ^d^
	Shatianyu	33.24 ± 0.01 ^i^	−0.5 ± 0.01 ^f^	1.48 ± 0.01 ^c^
HP juices	Grapefruit	38.40 ± 0.02 ^c^	4.31 ± 0.02 ^a^	7.55 ± 0.03 ^a^
	Guanxiyu	39.47 ± 0.02 ^b^	−1.23 ± 0.02 ^h^	−0.053 ± 0.01 ^f^
	Wendanyu	35.81 ± 0.02 ^g^	−1.48 ± 0.03 ^i^	1.56 ± 0.02 ^d^
	Liangpinyu	32.84 ± 0.02 ^j^	−0.73 ± 0.02 ^g^	2.66 ± 0.04 ^b^
	Pingshanyu	37.98 ± 0.01 ^d^	0.85 ± 0.01 ^d^	1.89 ± 0.01 ^c^
	Shatianyu	45.27 ± 0.01 ^a^	−1.57 ± 0.02 ^j^	0.93 ± 0.02 ^e^

Data are expressed as means ± standard deviation (*n* = 3); different letters indicate significantly different means at *p* < 0.05.

**Table 5:** Effects of cultivars on flavonoid composition of pomelo juices.

Types	Cultivars	Narirutin (mg L^−1^) y = 16191678.7116x + 296198.99829 (R^2^ = 0.999)	Naringin (mg L^−1^) y = 13268006.79 + 190986.41 (R^2^ = 0.999)	Hydroxynaringenin (mg L^−1^) y = 34294757.82x + 1610605.07 (R^2^ = 0.999)	Naringenin (g L^−1^) y = 23910089.51x + 1094130.87 (R^2^ = 0.999)	Sinensetin (g L^−1^) y = 15534697.16x + 398342.37 (R^2^ = 0.999)	Hesperidin (g L^−1^) y = 18934028.40x + 3184162.71 (R^2^ = 0.999)
LP juices	Grapefruit	140.11 ± 1.23 ^d^	519.10 ± 1.26 ^b^	-	1.19 ± 0.00 ^d^	0.23 ± 0.025	-
	Guanxiyu	25.70 ± 1.88 ^j^	75.90 ± 5.72 ^e^	427.52 ± 1.13 ^g^	1.17 ± 0.00 ^f^	-	-
	Wendanyu	121.55 ± 0.68 ^g^	-	452.36 ± 0.00 ^e^	1.08 ± 0.00 ^h^	1.30 ± 0.27	-
	Liangpinyu	200.10 ± 9.07 ^b^	-	452.36 ± 0.00 ^e^	1.18 ± 0.00 ^e^	-	1.53 ± 0.0018
	Pingshanyu	107.69 ± 0.00 ^h^	-	426.24 ± 0.00 ^g^	1.06 ± 0.00 ^i^	-	-
	Shatianyu	43.37 ± 4.99 ^i^	75.70 ± 4.18 ^e^	443.03 ± 0.00 ^f^	1.20 ± 0.00 ^c^	-	-
HP juices	Grapefruit	150.79 ± 1.85 ^c^	1109.4 ± 9.72 ^a^	-	1.20 ± 0.00 ^c^	0.22 ± 0.00	-
	Guanxiyu	107.68 ± 0.00 ^h^	133.3 ± 1.91 ^c^	462.35 ± 1.18 ^b^	1.22 ± 0.00 ^a^	-	-
	Wendanyu	142.88 ± 0.00 ^d^	-	465.22 ± 0.00 ^a^	1.19 ± 0.00 ^d^	1.30 ± 0.27	-
	Liangpinyu	689.32 ± 2.16 ^a^	-	464.48 ± 0.00 ^c^	1.21 ± 0.00 ^b^	-	1.61 ± 0.00
	Pingshanyu	138.12 ± 0.00 ^e^	-	451.58 ± 1.65 ^e^	1.15 ± 0.01 ^g^	-	-
	Shatianyu	131.57 ± 3.77 ^f^	100.3 ± 9.48 ^d^	459.52 ± 2.52 ^d^	1.21 ± 0.00 ^b^	-	-

Data are expressed as means ± standard deviation (*n* = 3); different letters indicate significantly different means at *p* < 0.05.

## Data Availability

Data is contained within the article.
